# Development of a Theoretical Continuous Glucose Monitoring Module for Pharmacy Students: Preparing Pharmacists for the Future

**DOI:** 10.3390/pharmacy12050154

**Published:** 2024-10-08

**Authors:** Florian Kinny, Bushra Ali Sherazi, Armin Dabidian, Stephanie Laeer, Emina Obarcanin

**Affiliations:** 1 Institute of Clinical Pharmacy and Pharmacotherapy, Heinrich-Heine University Duesseldorf, Universitaetsstr. 1, 40225 Duesseldorf, Germany; 2 Institute of Pharmacy, Faculty of Pharmaceutical and Allied Health Sciences, Lahore College for Women University, Lahore 54000, Pakistan; 3 Lee Kong Chian School of Medicine, Nanyang Technological University Singapore, 11 Mandalay Road, Singapore 308232, Singapore

**Keywords:** mobile health (mHealth), wearables, continuous glucose monitoring (CGM), digital literacy, digital competence, pharmaceutical care, education

## Abstract

To enhance the digital competencies of pharmacy students, the Institute of Clinical Pharmacy and Pharmacotherapy at Heinrich-Heine University Duesseldorf developed and evaluated a theoretical module on digital health and data analysis. This innovative module integrated a continuous glucose-monitoring (CGM) wearable device into teaching, providing students with in-depth practical experience and a 2.5 h seminar on digital health and CGM systems. Students’ knowledge of CGM and self-assessment of their CGM competencies were assessed in a pre-post manner. Additionally, students’ satisfaction with the module and their perceptions of the future integration of digital health training and the role of wearables in pharmacy practice were also assessed after the module. A total of 39 final-year pharmacy students completed the module conducted in June 2024 as part of the clinical pharmacy seminar. In total, 32 students completed the pre- and post-knowledge tests and self-assessment questionnaires. Both the knowledge and the students’ self-assessment of CGM-related skills after the module increased significantly (*p* < 0.05). Students expanded their knowledge regarding digital health solutions, in particular the CGM systems, and increased their self-reported competence in CGM-related skills. With this module, an important foundation was laid, as this is the first theoretical module including the essentials of CGM digital health tools for pharmacy students in Germany.

## 1. Introduction

In recent years, there has been a revolution in the usage of digital health tools and digital healthcare delivery with continuous progress at a rapid pace. Different domains of digital health such as electronic health (eHealth), mobile health (mHealth), telehealth, etc., are now being increasingly utilized in most medical and pharmaceutical fields to improve patient care [1,2]. The COVID-19 pandemic in particular has contributed to this development process with a major boost. Telemedical interventions, telepharmaceutical counseling, and remote patient monitoring have been widely available ever since [3], driving healthcare delivery improvement and reducing inequalities. However, digital tools must be used correctly, requiring a certain level of expertise. Hence, the continuous development in the field of digital health is not free of challenges [4]. Thus, the data collected must be reliable, secure, and certified by the relevant authorities, making the device collecting the data a medical device [5]. In addition, ethical and data protection aspects need to be taken into account. In order to fully realize the potential of novel technologies, these applications must be used widely, requiring seamless integration into healthcare processes, interoperability of all parties, and corresponding infrastructure by the regulatory authorities. In the specific field of mHealth, which is increasingly patient-centered, patients are empowered to take charge of their own health. 

Within the wide scope of digital health, patients can use mHealth tools, so-called wearable devices (wearables), to generate their own health data (patient-generated health data, PGHD), which can be used to monitor vital parameters, current therapy status, or chronic disease [6]. This trend in wearables use in patient care is expected to increase, making an immense amount of health data available. These data do not have to be therapeutically related but can also be collected individually to track exercise or someone’s personal daily life [7,8,9]. Wearables offer the advantage of more accurate documentation of health data and provide a comprehensive picture of health status. Such digital tools can make data from different life situations readily available, for example, sleep data. Another major advantage is the structured presentation of health data, which can be promptly analyzed and interpreted.

An example of such devices is the continuous glucose-monitoring (CGM) system. Consisting of a sensor, transmitter, and receiver, the system can display the current glucose concentration in the interstitial fluid (ISF) to the user each minute based on an electrochemical reaction [10]. The minimally invasive sensor is usually worn on the back of the upper arm for 14 days to collect several thousand data points during the wearing period, enabling the display of a comprehensive daily glucose profile, including measurements during the night. Self-monitoring of blood glucose (SMBG) is considered cumbersome and painful [11], and CGM has been shown to improve glycemic control in both Type 1 and Type 2 Diabetes Mellitus (T1DM and T2DM) [12,13]. There are ample reports regarding the broad use of CGM already to replace SMBG, including standards of care documents and registry reports [14]. CGM can reduce both the HbA1c values and hypoglycemic periods in T1DM patients [15,16,17]. Based on the amount of glycemic data, further metrics can be calculated to describe the course of the disease. In addition to glucose variability (GV), the time in a target range (TIR) can also be quantified with these devices. Both parameters offer added value for therapy monitoring and show the limitations of HbA1c, which can only be seen as an average of the glucose data from the last 2–3 months. So far, in Germany, CGMs have been mainly used in the therapy and disease monitoring of insulin-dependent diabetes mellitus (IDDM). Since 2016, the costs of CGM devices have been covered by statutory health insurance (SHI) for patients with IDDM in Germany [18]. Due to SHI reimbursement the use of CGM has continuously been increasing since 2016, with more than two-thirds of children and adolescents with T1DM in Germany using CGM [19,20]. Similar examples are available from other countries, for instance, a study from Norway indicated increased usage of CGM from 34% in 2016 to 97% in 2022 among T1DM children and adolescents [14]. These devices are also commercially available and have been widely used in the non-therapeutic area for tracking personal lifestyle and diet. This increasing use of CGM inevitably leads to more readily available health data. It can be expected that the healthcare systems will increasingly make use of these data and will evaluate their added value to individual patients.

Pharmacists are seen as easily accessible and highly qualified players in the healthcare system [21]. They will be increasingly confronted with wearables and their valuable data in the future. Pharmacists have been shown to improve health outcomes through direct patient care [22]. The pharmaceutical care of CGM patients is also possible remotely and is conceivable in the future, especially in the light of increasing use of digital health technologies [23]. Since 2022, patients with SHI in Germany have been legally entitled to additional healthcare and pharmaceutical services, which are planned to be expanded in 2025 to include intensive counseling and measures carried out by pharmacies for the prevention and early detection of diabetes mellitus [24]. It is anticipated that different diabetes technologies including CGM will be discussed during such pharmaceutical consultations. Therefore, dealing with this topic and the large amount of data requires pharmacists to have a certain level of digital competencies [25], especially since a lack of digital literacy has already reported [26,27].

To meet these growing requirements, digital competencies need to be taught and acquired not only by practicing pharmacists but also by pharmacy students during their studies. Therefore, a team of researchers at the Institute of Clinical Pharmacy, Heinrich-Heine University Duesseldorf developed and evaluated a theoretical module on teaching and analyzing digital content with CGM data. We hypothesize that the implementation of a theoretical module in the pharmacy curriculum is beneficial to prepare pharmacy students for future practice challenges regarding digital health.

## 2. Materials and Methods

### 2.1. Study Design and Participants

**Figure 1:**
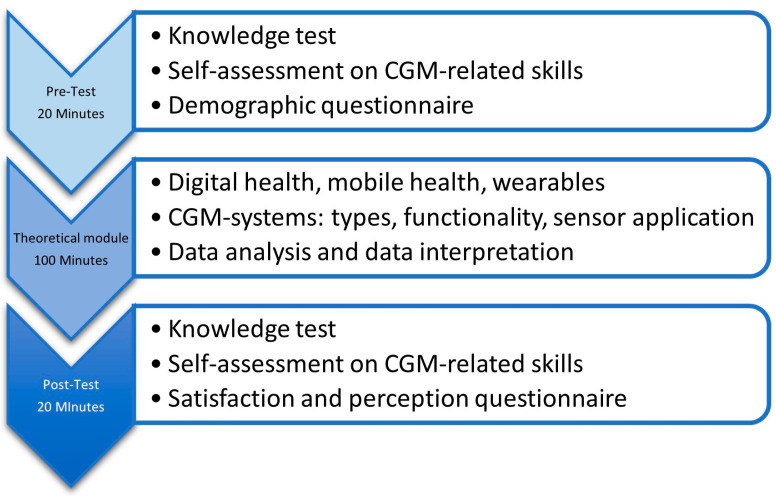
Flowchart of the study design. CGM = continuous glucose monitoring.

This research project aimed to investigate the change in knowledge score and the self-assessment of final-year pharmacy students’ competencies in “Digital health: handling wearables data” using a pre- and post-design (Figure 1). A corresponding module was conducted as part of the “Clinical Pharmacy” seminar at the Heinrich-Heine-University Duesseldorf in June 2024. The participating pharmacy students had the opportunity to give their consent to the processing of study-related data after they had been able to read and understand the study information. Study data were collected directly in anonymized form. To be able to compare the pre-test scores with the post-test scores of individual participants, each participant created an individual, anonymous study code at the beginning of the tests by answering questions about their social life (e.g., “What is the first letter of your mother’s name?”, “How many siblings do you have?”). Previous experience with a CGM device was an exclusion criterion for participation in the knowledge test and self-assessment survey. Approval for this study was granted by the ethics committee of the medical faculty of Heinrich-Heine University Duesseldorf (Nr. 2024-2834). It has been registered in the German clinical trial registry (Nr. DRKS00034891).

### 2.2. Training and Education Module

The training and education module was conducted over 2.5 h by an academic faculty member. Firstly, a general overview of digital health, its areas of application, and potentials and challenges were presented. References and comparisons were drawn regarding the current development of digital transformation in the German healthcare system. The module included examples of recent digitalization and common digital tools used in the German healthcare system, starting with an insight into the electronic health card and the electronic health records (EHRs) for storing relevant patient information, and its ability to redeem electronic prescriptions. The module provided insights into the use of artificial intelligence (AI) in the healthcare sector. Data analysis using machine learning (ML) was briefly described and the importance of healthcare data and data protection was discussed. Furthermore, the main topic of mhealth including the CGM system and its generated data were discussed, as well as setting up the system app, the correct application, general functionality, handling, and the difference between various CGM device types. The indications for real-time-CGM (rt-CGM) devices and the advice to customers on purchasing the device and the reimbursement process by SHI were explained. The advantages of CGM over SMBG were listed, and the corresponding evidence was cited. All data, reports, and metrics of CGM data related to the international consensus [28] were then presented and discussed in detail. In addition, students were shown how to analyze and interpret these data using an anonymous Ambulatory Glucose Profile (AGP) report. Factors influencing glucose levels such as food, food composition, and quantity, order of food intake, exercise, stress, and menstrual cycle were explained. A patient case was presented and discussed. The pharmacist faculty member guided the participants through the AGP report and its analysis. Finally, the participants were asked to discuss a T2DM patient case by evaluating the data, making a treatment recommendation, and advising the patient on lifestyle changes.

### 2.3. Instruments for Student Assessment

#### 2.3.1. Knowledge Test

An online knowledge test of 12 questions was created to investigate the students’ knowledge of the topic of CGM and the associated data (Supplementary Materials). The questions, being marked as multiple-select or single-answer questions, were based on a questionnaire created by Sherrill et al., which was modified and expanded by members of the faculty [29]. The questions were about CGM functionality, CGM data analysis, CGM data interpretation, and making recommendations about therapy and lifestyle changes. Participants were able to access the electronic questionnaire for both the pre-test and the post-test via a quick response (QR) code. Demographic data such as gender and age were also collected once during the pre-test.

#### 2.3.2. Self-Assessment Questionnaire

The self-assessment of the participants’ competence on the topic of CGM and the associated data were also carried out in both the pre- and post-test using a seven-point Likert scale, where one stands for “full disagreement” and seven stands for “full agreement” (Supplementary Materials). The questionnaire consisted of five statements relating to CGM-related skills: sensor application, advising patients on how the CGM system works and how to use it, analyzing CGM data, making treatment recommendations to a physician, and advising patients on any lifestyle changes. This questionnaire was developed by the module coordinator and reviewed by two other faculty members. 

#### 2.3.3. Satisfaction and Perception Questionnaire

At the end of the module, the participants were asked about their satisfaction with the module by using a slider bar, with a minimum value of 0 representing “very dissatisfied” and a maximum value of 100 representing “very satisfied” (Supplementary Materials). Moreover, two additional statements about student perceptions of integrating digital health training and education during pharmacy studies and the role of wearables in future pharmacy practice were also rated on a seven-point Likert scale of agreement, where one stands for “full disagreement” and seven stands for “full agreement” (Supplementary Materials).

### 2.4. Statistical Analysis

In this study, the change in knowledge and self-assessment scores of final-year pharmacy students before and after the module was investigated. To compare the change in knowledge and self-assessment scores from the pre-test to the post-test, a paired sample t-test was used as a parametric test. The significance level alpha was set to 0.05. Descriptive statistics were used to describe the demographic data. Microsoft Excel^®^ [30] and R-Programming language [31] were used to handle the data. OriginPro 2021^®^ [32] was used to statistically analyze the data. QualtricsXM^®^ software [33] was used to create and administer the electronic questionnaires.

## 3. Results

A total of 39 final-year pharmacy students took part in this module and provided their consent to collect and process their study-related data. Out of these, 32 answered both the pre-and post-test. Four students were excluded due to their previous CGM experience. A further three students were excluded from the data analysis due to missing data. Table 1 shows the demographic data of the participants.

### 3.1. Knowledge Test

**Figure 2:**
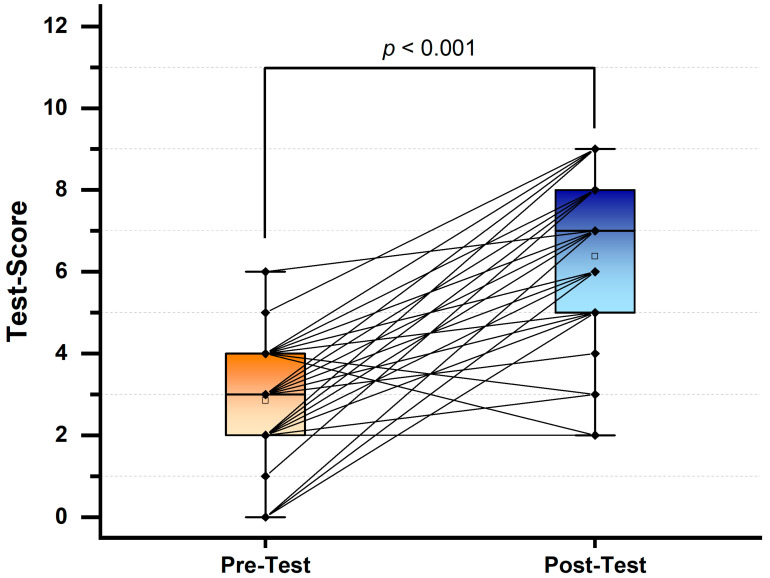
Box plots of final-year pharmacy students’ pre- and post-test scores. The black dots and lines represented the difference in performance of each student. The hollow box represents the mean value. A paired *t*-test with a significance level of alpha = 0.05 was used to compare the test scores.

On a 12-point scale, the participants’ CGM knowledge improved significantly from the pre- to post-test, indicated as a mean with standard deviation (SD) for the pre-test score of 2.84 ± 1.42 compared to the post-test score of 6.38 ± 1.96 (*p* < 0.05). A large majority of participants (90.6%, 29/32) achieved a higher score in the post-knowledge test scores compared to their pre-test scores. Only one participant had the same pre-post scores, whereas two participants had a lower score in the post-test than in the pre-test (Figure 2).

### 3.2. Self-Assessment

**Figure 3:**
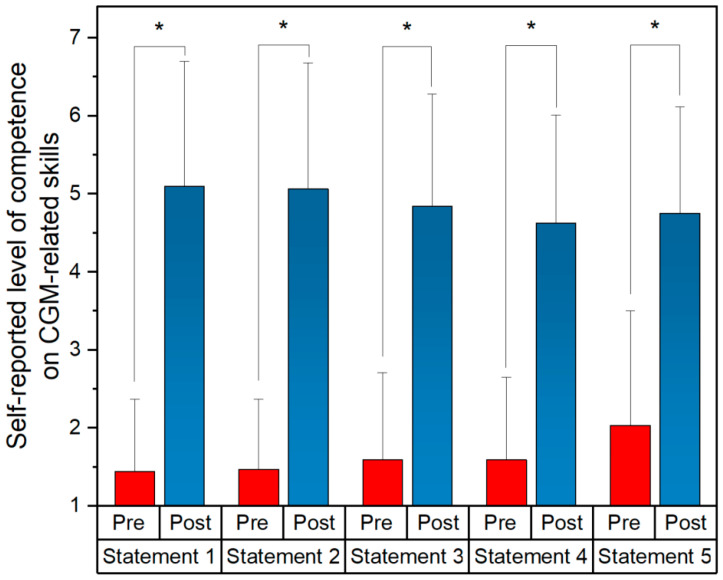
Bar plots of mean values, with the error bar representing standard deviation (SD) of self-assessment scores on CGM-related skills for pre- and post-questionnaire using a seven-point Likert scale. * *p* < 0.05 indicating statistical significance; CGM = continuous glucose monitoring; n = 32.

Overall, participating pharmacy students rated their digital competence on CGM-related skills in post self-assessments significantly higher than those in the pre-questionnaire (Table 2, Figure 3). None of the students rated themselves worse after the module in the post-questionnaire compared to the pre-questionnaire. Each of the five statements were rated significantly higher by the 32 pharmacy students (*p* < 0.05). The mean values with a 95% confidence interval (CI) of all statements before the module were within the range of “disagreement”. After the module, the mean values of all statements including the 95% CI were within the “agreement” range.

### 3.3. Satisfaction and Perception Questionnaire

**Figure 4:**
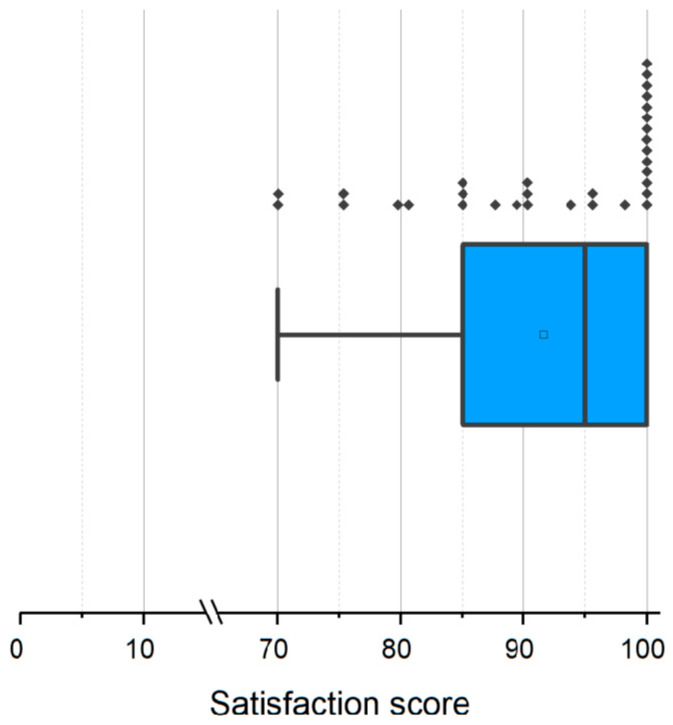
Box plot with the distribution of student’s satisfaction with the module collected with questionnaire (0 = “very unsatisfied”, 100 = “very satisfied”). Results ranging from 70 to 100 with a median of 95 and a mean (±standard deviation) of 91.59 (±9.68).

Participants declared high satisfaction as rated using a slider bar ranging from 0 (“very unsatisfied”) to 100 (“very satisfied”). On average, the module was rated as very satisfactory, with a mean of 91.59 points ranging between 70 and 100 points (Figure 4). Out of the 32 participating pharmacy students, 14 (43.75%) awarded maximum points for the module.

**Figure 5:**
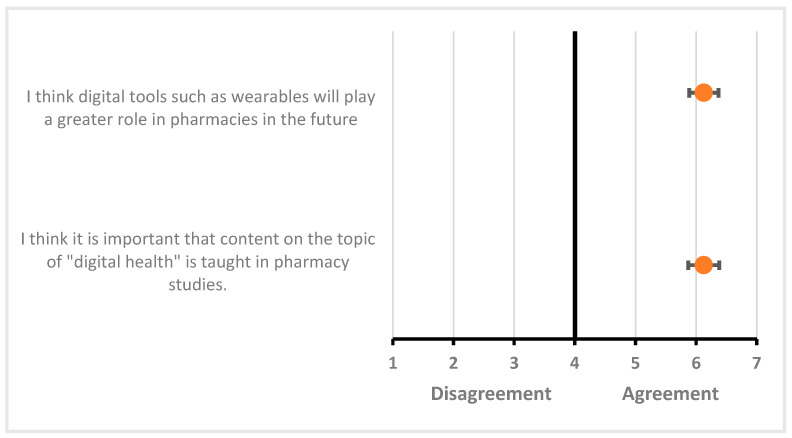
Forest plot of mean values with 95% confidence interval (CI) of perception questionnaire scores (seven-point Likert scale); n = 32 (1 = “very strongly disagree”, 2 = “strongly disagree”, 3 = “disagree”, 4 = “neither agreeing nor disagreeing”, 5 = “agree”, 6 = “strongly agree”, 7 = “very strongly agree”).

Using a seven-point Likert scale, participants were able to assess two statements on the integration of digital health in teaching and in community pharmacy practice. They considered the teaching of content on the topic of “digital health” to be important (6.13 (±0.7)) and thought that wearables would play an important role in community pharmacy practice in the future (6.13 (±0.74)). Responses are indicated as mean values with 95% CI (Figure 5).

## 4. Discussion

To the authors’ knowledge, this is the first study in Germany evaluating the effectiveness of a module that integrates theoretical content on a CGM device and analysis of related health data into pharmacy education. In this study, final-year pharmacy students’ knowledge of CGM devices and CGM data analysis increased significantly after attending the module. It could be demonstrated that students achieved higher self-assessment scores of competencies to CGM-related skills, resulting in a significant increase from pre- to post-questionnaire. The participants had high levels of satisfaction with the module and had positive perceptions about the integration of digital health content into pharmacy curricula and the future role of pharmacists around wearables. 

In times of increasing digitalization in the healthcare system, the growing number of wearables, and the availability of a large amount of health data [9], pharmacists, being easily accessible and well-qualified players in the healthcare system [21], need to be trained and prepared for this transformation as soon as possible. In Germany, a draft law plans to expand the five existing pharmaceutical care services to also include intensive diabetes counseling by 2025 [24]. It is expected that CGM data will be widely used in these consultations, highlighting the urgent need to prepare future pharmacists for handling and interpreting these data, a task currently completely missing in pharmacy education. With this research project, we were able to show that the implementation of a theoretical module is possible and has a positive effect on both knowledge and self-assessed digital competency improvements.

The presented method of knowledge transfer seems effective, given that 29 of the 32 participants were able to improve their pre-test score after the teaching intervention, with only three participants not improving their test score, corresponding to 9.38% of total participants for whom the teaching method used had no effect. The increase in knowledge of 3.54 test points on average is also comparable with other studies in the literature. In a study about education in CGM for pharmacists and pharmacy students, Sherrill et al. developed and evaluated a hands-on training program [29]. Over two weeks, practical and theoretical content relating to a CGM device was taught under the guidance of two pharmacists. The associated seminars covered content on lifestyle and eating habits, as well as the assessment of CGM reports with corresponding data analysis. Using their objective knowledge test of 10 questions about CGM, the authors were able to determine a significant increase in knowledge from pre-test 4.1 to post-test 7, indicated as mean values. However, the comparability of the results is limited, as the intervention consisted not only of theoretical content but also of practical experience with the wearable.

Using a self-assessment questionnaire, it was found that participants considered themselves more competent in terms of CGM counseling and its data after the intervention. Each CGM-related skill of the questionnaire was rated significantly higher after the intervention. The lowest value was reported for the pre-survey item “sensor application”. This could be explained by the fact that pharmacy teaching in Germany is not patient-oriented. These results are consistent with those of a previous pilot study in which Kinny et al. were able to show that the theoretical and concurrent practical teaching of digital health content can improve students’ self-assessment on CGM-related skills. Under the supervision of two faculty members, four students were introduced to two wearables over a period of 14 days and analyzed the health data collected with them [34]. However, the small number of participants in the previous pilot study does not allow a general conclusion. In addition, this present study did not include practical handling nor instructions about CGM use and application. The associated costs, time, and personnel efforts in practical wearable courses are substantial and present pharmacy schools with great challenges. Establishing a theoretical module, as presented in this paper, can help overcome these hurdles and impart important knowledge for future pharmaceutical counseling.

The participants had positive perceptions about the integration of digital health content into pharmacy curriculum and the future role of pharmacists in the area of wearables. The results highlight the urgent need for applied teaching of important digital health tools and technologies in pharmacy and underline the awareness of the emerging challenges for providing consultation using digital tools such as wearables among pharmacy students. Our result is consistent with previous studies with pharmacy students; Darnell et al. examined the change in attitudes towards digital health among pharmacy students in a pre-post procedure. Using the Digital Health Familiarity, Attitudes, Comfort, and Knowledge Scale (DH-FACKS) as a validated questionnaire, a significant increase in scores among pharmacy students was observed following their participation in a year-long discussion-based case conference series focused on digital health topics [35].

The participants expressed a high level of satisfaction with the module, expressed via a slider bar of 0–100, with the lowest value being 70, while 43.75% of the participants rated this module with the highest value of 100. This result could be explained by the high level of interest among participants in the topic of digital health, which was demonstrated in an international survey among universities and pharmacy schools [25]. With a mean age of 25.69 years, the participants in the study were young and more likely to be familiar with digital tools and software, which underlined their interest in the digital topic.

It has repeatedly been reported that there is a need to prepare pharmacists for the ongoing digital transformation in healthcare [25,36,37,38]. In 2022, Knezevich et al. conducted a survey of US universities and colleges to investigate whether at what point in the curriculum and to what extent teaching on CGM is provided. They found that 89% of the schools surveyed teach CGM for a median of one hour. However, the amount, timing, and methodology of such teaching interventions need to be further investigated, which will increase the need for teaching [39]. 

Concepts for the integration of digital health in teaching are known in various health disciplines which are feasible and being described in the literature [40,41,42,43,44]. The implementation of those modules in Germany is feasible. For example, Aulenkamp et al. were able to demonstrate the successful development of a corresponding course on digital health for medical students in Germany [45]. The handling of health data and the teaching of this content to pharmacists continues to pose a significant challenge in Germany, among other countries [25]. There is already evidence of how digital health content can be added to the pharmacy curricula [46,47,48]. Obarcanin et al. summarize the contents of a multinational workshop and present ways in which corresponding concepts can be implemented in teaching for pharmacists as well as other health disciplines [49]. In addition, the first modules have already been implemented in pharmacy studies in Germany [34,50]; however, further systematic implementation and integration in the pharmacy curriculum is needed to face the challenges of the upcoming digital age. The Institute of Clinical Pharmacy and Pharmacotherapy, Heinrich-Heine University Duesseldorf, has already undertaken the integration of digital health content for training pharmacy students and would strive to expand it by implementing didactic and experiential learning. For this purpose, a digital health lab has been established. Pharmacy students are trained for different patient care scenarios using high-fidelity simulators [51,52] for telepharmacy-based consultations [53], with diabetes mobile health apps and wearables [34,54].

We are aware of some of the limitations of our study. Firstly, since we only measured knowledge and self-assessment before and immediately after the intervention, no data are available over a longer period. It is therefore not possible to predict whether the increase in knowledge score will persist after a longer period. Studies regarding an effect over a longer period were outside the scope of this project. Furthermore, although the participants’ knowledge was assessed using an objective test, the participants’ competence in CGM-related skills was only measured using subjective self-assessment questionnaires. For future studies, CGM-device and data-specific skills can be investigated using objective assessments such as an objective structured clinical examination (OSCE). However, the associated time and personnel constraints limited the possibilities of this 2.5 h seminar. In addition, the transfer of knowledge was based solely on theoretical content without any practical experience. We believe, that combining theoretical and practical modules in a teaching program like this could improve the quality of knowledge transfer. Furthermore, participants could use their own wearable to access and learn from their data, with the limitation of high costs of wearable devices. Nevertheless, there is a high demand for the communication and education of content on the topic of digital health tools and technologies.

## 5. Conclusions

In times of advancing digitalization in the healthcare sector and the increasing use of wearables and their data, pharmacists as easily accessible healthcare professionals are expected to assume a central role. Therefore, pharmacists need to be prepared for digital transformation during their studies and before entering professional pharmacy practice. The long-needed and necessary implementation of a theoretical module in CGM for student pharmacists is feasible as shown by the findings of our study. A comprehensive, systematic theoretical and practical training approach will be required to meet the growing necessity of digital health in pharmacy education.

## Figures and Tables

**Table 1:** Participant characteristics.

Participants recruited	*n* = 39
excluded	*n* = 7 missing data = 3 previous CGM experience = 4
Participants included in the analysis	*n* = 32
Age	
Mean (±SD) Median Range	25.69 (±2.44) 23.5 21–29
Gender	
Female, *n* (%) Male, *n* (%)	25 (78.125) 7 (21.875)

SD = standard deviation; CGM = continuous glucose monitoring.

**Table 2:** Questions of the self-assessment questionnaire.

ID	Statement	Pre-Questionnaire Mean (±SD)	Post-Questionnaire Mean (±SD)	*p*-Value
Statement 1	I feel competent to apply a CGM system to a patient	1.44 (±0.93)	5.09 (±1.61)	<0.001
Statement 2	I feel competent to advise a patient on how their CGM system works and how to use it	1.47 (±0.9)	5.01 (±1.62)	<0.001
Statement 3	I feel competent in analyzing CGM data	1.59 (±1.11)	4.83 (±1.44)	<0.001
Statement 4	I feel competent to suggest therapy adjustments to the doctor based on CGM data	1.59 (±1.06)	4.63 (±1.39)	<0.001
Statement 5	I feel competent to make therapy and lifestyle recommendations to the patient based on CGM data	2.03 (±1.47)	4.75 (±1.37)	<0.001

SD = standard deviation, CGM = continuous glucose monitoring (1 = “very strongly disagree”, 2 = “strongly disagree”, 3 = “disagree”, 4 = “neither agreeing nor disagreeing”, 5 = “agree”, 6 = “strongly agree”, 7 = “very strongly agree”).

## Data Availability

The dataset presented in this study is available from the corresponding author on reasonable request.

## Supplementary Materials

The following supporting information can be downloaded at https://www.mdpi.com/article/10.3390/pharmacy12050154/s1.
