# Supplementary material for: Development of a Theoretical Continuous Glucose Monitoring Module for Pharmacy Students: Preparing Pharmacists for the Future
Source: Pharmacy (Basel). 2024 Oct 8;12(5):154. doi: 10.3390/pharmacy12050154 (PMC11511089; doi:10.3390/pharmacy12050154)
Supplement: Supplementary file 1 [file pharmacy-12-00154-s001.zip › pharmacy-3185107-supplementary.pdf]

1. Demographics

How old are you?

What's your gender?

Have you already had experience with a CGM system? A) Yes B) No

2. CGM-Knowledge-Test (Pre- and Post-test)

Answer the following questions:

1.1 Where does glucose measurement take place in CGM systems? (single answer)

A In a very fine blood vessel

B In the muscle cells

C In the subcutaneous fatty tissue

D In the upper skin cells

1.2 What should the patient most likely do if the glucose value rises steeply half an hour after breakfast and the trend arrow is pointing upwards? (multiple answers possible)

A To avoid hyperglycaemic events, give an additional 2 units of insulin as a precaution

B Increase the basal insulin by 20% for the next 2 hours

C Omit the next snack

D Correct at the earliest 2 hours after the meal and consider the insulin that is still effective

1.3 Which statement(s) regarding the evidence of the CGM system is/are true? (multiple answers possible)

A Glucose variability is a predictor of hypoglycemia

B Time on target measured by CGM is associated with diabetic retinopathy in type 2 diabetics, but not associated with diabetic neuropathy

C The metrics collected by CGM systems can replace the HbA1c value

D The use of CGM can reduce HbA1c and glucose variability

1.4 Which statement(s) about the calibration of a CGM device by the patient is/are true? (multiple answers possible)

A Not all CGM devices need to be calibrated

B Every CGM device must be calibrated once a day by measuring the blood glucose level

C Every CGM device must be calibrated once a week by measuring the blood glucose level

D Calibration must always be carried out before sporting activity

1.5 Which statement(s) regarding the recommendations according to international consensus for target values in the target range for a 28-year-old type 2 diabetic is/are NOT true? (multiple answers possible)

A It is recommended to set the target range at 70-180 mg/dL

B It is recommended that at least 85% of the time be spent in the set target range

C The recommendation provides for a narrower hypoglycemic range compared to the hyperglycemic range.

D During pregnancy it is recommended to increase the target range

1.6 What information does the line graph in the Ambulatory Glucose Profile of CGM data provide? (multiple answers possible)

A It shows the times of the day when the values are particularly high or particularly low

B It shows the average glucose value of the last week

C It shows whether the blood glucose value has fluctuated greatly

D The HbA1C can be derived from the lines

1.7 How can the following sentence be completed correctly? (multiple answers possible)

High glucose variability (GV)...

A ...is often accompanied by a short time in the target range

B ...does not influence the time in the target range

C ...is recognizable in the Ambulatory Glucose Profile (AGP)

D ...is not associated with the occurrence of hypo- and hyperglycemia

1.8 A 55-year-old type 2 diabetes mellitus patient comes to your pharmacy. She tells you about her current medication, which consists of basal insulin (35 I.U.) and prandial insulin (12 I.U.) three times a day. Because it hurts, she does not measure her blood glucose levels consistently. She asks whether there is an alternative to finger pricking. In this case, would the costs of a CGM system be covered by statutory health insurance (SHI)? (single answer)

A No, only type 1 diabetics can claim the costs of a CGM system from the SHI

B Yes, as an insulin-dependent type 2 diabetic with intensified insulin therapy , she is entitled to claim

C No, because the patient is over 50 years old

D Yes, because the patient does not carry out blood glucose measurements by finger prick.

1.9 Kim is a 35-year-old woman who has recently been diagnosed with type 2 diabetes mellitus with an HbA1c of 13.2%. She was switched to an insulin regimen consisting of basal insulin once daily and bolus insulin three times daily before each meal. Continuous glucose monitoring shows a periodic sharp rise in blood glucose levels from 55-60 mg/dL to over 200 mg/dL. What is the most likely explanation for this drastic change in blood glucose levels?

A Kim is overdosing her bolus insulin

B Kim underdoses her basal insulin

C Kim is overtreating hypoglycemia

D Kim exercises vigorously

1.10. Mr. Meier is 45 years old, has type 2 diabetes mellitus and no other significant medical history. He currently takes a basal insulin once a day and a bolus insulin three times a day before each meal. His HbA1c level is 6.9%. A continuous glucose meter shows typical glucose levels of 80-90 mg/dL before breakfast, 120-140 mg/dL before lunch, 160-180 mg/dL before dinner, 200-220 mg/dL before bedtime and 50-55 mg/dL overnight. Which of the following would be most appropriate in relation to Mr. Meier's insulin regimen? (single answer)

A Make no changes, as Mr. Meier's HbA1c level has reached the target of less than 7% for an otherwise healthy 45-year-old

B Increase bolus insulin as glucose levels rise steadily throughout the day

C Maintaining the current basal insulin dose, as the glucose level before breakfast has reached the target of 80-130 mg/dL

D Reducing the basal insulin dose and increasing the bolus insulin dose due to his current glucose spikes

1.11. Which statement(s) is/are true? (multiple answers possible)

A Glucose variability expressed as a coefficient of variation should be less than 5% according to international consensus

B A wide 5-95 percentile range in the line graph of the AGP shows glycemic variations that are mainly caused by the patient's lifestyle.

C A wide 25-75 percentile range in the line graph of the AGP shows glycemic variations mainly caused by the patient's lifestyle

D The glucose management indicator (GMI) can be used to estimate the HbA1C value

1.12. Which statement(s) on the development of an Ambulatory Glucose Profile (AGP) is/are true?

(multiple answers possible)

A Times in, above and below the target range are shown as a stacked bar chart

B It shows glucose statistics such as glucose management indicator or glucose variability

C It indicates the period in which the sensor has been active

D Individual daily profiles of the analysis period are displayed

3. Self-assessment of competence with regard to consulting on CGM device and data (Pre- and Post-Test)

<u>How would you rate yourself with regard to the following statements? Use a seven-point Likert-scale:</u>

1 = Very strongly disagree
2 = Strongly disagree
3 = Disagree
4 = Neither agreeing nor disagreeing
5 = Agree
6 = Strongly agree
7 = Very strongly agree

3.1 I feel competent to apply a CGM system to a patient

3.2 I feel competent to advise a patient on the functioning and use of their CGM system

3.3 I feel competent to analyze CGM data

3.4 I feel competent to suggest therapy adjustments to the doctor based on CGM data

3.5 I feel competent to make therapy & lifestyle recommendations to the a patient based on CGM data

4. Satisfaction-questionnaire (Post-questionnaire)

<u>How satisfied have you been with the module?</u>

0 = very unsatisfied

100 = very satisfied

5. Perception-questionnaire (Post-questionnaire)

<u>How would you rate yourself with regard to the following statements? Use a seven-point Likert-scale:</u>

1 = Very strongly disagree
2 = Strongly disagree
3 = Disagree
4 = Neither agreeing nor disagreeing
5 = Agree
6 = Strongly agree
7 = Very strongly agree

5.1 It is important that digital health content is covered in pharmacy curricula.

5.2 Digital tools such as wearables will play a greater role in pharmacies in the future.

ID	Statement	Pre-Questionnaire Mean (±SD)	Post-Questionnaire Mean (±SD)	***p*-value**
Statement 1	I feel competent to apply a CGM system to a patient	1.44 (±0.93)	5.09 (±1.61)	**< 0.001**
Statement 2	I feel competent to advise a patient on how their CGM system works and how to use it	1.47 (±0.9)	5.01 (±1.62)	**< 0.001**
Statement 3	I feel competent to analyze CGM data	1.59 (±1.11)	4.83 (±1.44)	**< 0.001**
Statement 4	I feel competent to suggest therapy adjustments to the doctor based on CGM data	1.59 (±1.06)	4.63 (±1.39)	**< 0.001**
Statement 5	I feel competent to make therapy and lifestyle recommendations to the patient based on CGM data	2.03 (±1.47)	4.75 (±1.37)	**< 0.001**

ID	Statement	Pre-Questionnaire Mean (CI)	Post-Questionnaire Mean (CI)	***p*-value**
Statement 1	I feel competent to apply a CGM system to a patient	1.44 (0.32)	5.09 (0.56)	**< 0.001**
Statement 2	I feel competent to advise a patient on how their CGM system works and how to use it	1.47 (0.31)	5.01 (0.56)	**< 0.001**
Statement 3	I feel competent to analyze CGM data	1.59 (0.39)	4.83 (0.5)	**< 0.001**
Statement 4	I feel competent to suggest therapy adjustments to the doctor based on CGM data	1.59 (0.37)	4.63 (0.48)	**< 0.001**
Statement 5	I feel competent to make therapy and lifestyle recommendations to the patient based on CGM data	2.03 (0.51)	4.75 (0.47)	**< 0.001**

CGM = Continuous Glucose Monitoring; AGP = Ambulatory Glucose Profile; GV = Glucose Variability; dL = Deciliter; mg = Milligram; SHI = Statutory Health Insurance; GMI = Glucose Management Indicator; SD = Standard Deviation; CI = 95% Confidence Interval
